# Quantitative Control of Early Flowering in White Lupin (*Lupinus albus* L.)

**DOI:** 10.3390/ijms22083856

**Published:** 2021-04-08

**Authors:** Sandra Rychel-Bielska, Anna Surma, Wojciech Bielski, Bartosz Kozak, Renata Galek, Michał Książkiewicz

**Affiliations:** 1 Department of Genetics, Plant Breeding and Seed Production, Wroclaw University of Environmental and Life Sciences, 50-363 Wrocław, Poland; sandra.rychel-bielska@upwr.edu.pl (S.R.-B.); bartosz.kozak@upwr.edu.pl (B.K.); renata.galek@upwr.edu.pl (R.G.); 2 Department of Gene Structure and Function, Institute of Plant Genetics, Polish Academy of Sciences, 60-479 Poznań, Poland; surmaanna97@gmail.com (A.S.); wbie@igr.poznan.pl (W.B.)

**Keywords:** vernalization, markers, flowering, quantitative trait, selection

## Abstract

White lupin (*Lupinus albus* L.) is a pulse annual plant cultivated from the tropics to temperate regions for its high-protein grain as well as a cover crop or green manure. Wild populations are typically late flowering and have high vernalization requirements. Nevertheless, some early flowering and thermoneutral accessions were found in the Mediterranean basin. Recently, quantitative trait loci (QTLs) explaining flowering time variance were identified in bi-parental population mapping, however, phenotypic and genotypic diversity in the world collection has not been addressed yet. In this study, a diverse set of white lupin accessions (*n* = 160) was phenotyped for time to flowering in a controlled environment and genotyped with PCR-based markers (*n* = 50) tagging major QTLs and selected homologs of photoperiod and vernalization pathway genes. This survey highlighted quantitative control of flowering time in white lupin, providing statistically significant associations for all major QTLs and numerous regulatory genes, including white lupin homologs of *CONSTANS*, *FLOWERING LOCUS T*, *FY*, *MOTHER OF FT AND TFL1*, *PHYTOCHROME INTERACTING FACTOR 4*, *SKI-INTERACTING PROTEIN 1*, and *VERNALIZATION INDEPENDENCE 3*. This revealed the complexity of flowering control in white lupin, dispersed among numerous loci localized on several chromosomes, provided economic justification for future genome-wide association studies or genomic selection rather than relying on simple marker-assisted selection.

## 1. Introduction

White lupin (*Lupinus albus* L.) is an annual legume plant cultivated for animal feed and human consumption in Europe, Africa and Australia [1]. It is also a valuable component in crop rotation and organic farming, thanks to its nitrogen fixation via diazotrophic symbiosis and mobilization of soil phosphorus by formation of cluster roots [2,3,4]. White lupin seeds are a valuable source of protein (38–42% dry weight) and oil (10–13%) with advantageous omega-6 to omega-3 acid ratios [5,6,7]. Moreover, white lupin consumption has positive nutraceutical impact, reducing hyperglycaemia, hypercholesterolemia and hypertension [8]. For many years, wide use of lupins for human consumption was hampered by their bitter taste caused by a high alkaloid content, but this issue has been fixed in modern cultivars [9]. Considering the agronomic characteristics of white lupin, there is still high potential for further yield increases and drought tolerance improvement by exploitation of existing germplasm resources [10,11,12,13].

It is assumed that white lupin originated from the Balkan-Mediterranean area [14]. Native adaptation to environmental conditions of many lupin landraces is the requirement of a prolonged cold period, named vernalization, to initiate flowering. Thus, in late flowering and vernalization-responsive lines, time to flowering is reduced proportionally to the length of the vernalization period [15]. White lupin is currently grown under wide range of climatic conditions, including humid tropics, the Mediterranean basin as well as temperate zones with humid or intermediate continental climates. In some of these regions highly delayed flowering in the absence of vernalization is a very undesired trait.

Moreover, white lupin was revealed to be susceptible to anthracnose, a very devastating disease first reported in Brazil in 1912, then in the USA in 1939, and, finally, until 1997, it appeared in all major regions where white lupin was cultivated [16,17,18,19]. Unfortunately, major European early flowering white lupin donors were found to be very susceptible to anthracnose [20,21]. Substantial levels of resistance to anthracnose were found only in several accessions originating from mountainous regions of Ethiopia, particularly in lines P27174, P27175, and P27178 [22]. Marker-based studies revealed that Ethiopian white lupins are relatively closely related to each other and very distinct from the improved germplasm originating from Europe [23]. A reference anthracnose resistance donor, line P27174, was found to carry two major undesired traits, namely a high vernalization requirement and high alkaloid content, and as such was exploited, together with Kiev Mutant carrying opposite traits, for the development of mapping populations and the construction of the linkage map [24]. Following progress in molecular techniques, this linkage map was subsequently improved with novel markers [25,26]. The most recent version of the linkage map served as an anchor for the establishment of two genome sequence assemblies [27,28]. Quantitative trait loci (QTL) mapping, based on the same mapping population, revealed the presence of two major QTLs for anthracnose resistance, located in the linkage groups ALB02 and ALB04, as well as five major QTLs for flowering time, two of them located in the linkage group ALB02 (including one overlapping with the major anthracnose resistance locus), another two in the linkage group ALB13, and one in the linkage group ALB16 [24,25,26]. The white lupin molecular toolbox has been recently supplemented with PCR-based markers suitable for tracking Ethiopian alleles of major anthracnose resistance loci, alkaloid locus *pauper* and main candidate genes controlling flowering induction [29,30,31]. Interestingly, white lupin breeders revealed that joining of early flowering with anthracnose resistance in the progeny descending from the cross between Kiev Mutant and P27174 was ineffective, however, replacement of the Ukrainian donor of early flowering by the other (French) thermoneutral germplasm made this process much more feasible [22,32]. Such an observation highlighted the hypothetical presence of additional genes controlling early flowering in white lupin collection, genetically different than those revealed in population mapping.

Indeed, the lack of knowledge on distribution of alleles controlling flowering time in worldwide white lupin germplasm collection hampers selection of compatible early flowering germplasm for crosses in current breeding approaches. The present study aimed to address this issue by combining phenotyping of time to flowering in diversified germplasm collection with genotyping of marker alleles across all major QTL loci conferring flowering time control as well as some candidate genes from flowering regulation pathways. This approach provided novel evidences for quantitative regulation of flowering induction in white lupin and designated novel candidate donors of earliness and thermoneutrality for further improvement of this species as a crop.

## 2. Results

### 2.1. Early Flowering and Thermoneutrality Is Present in Primitive and Domesticated Germplasm

**Figure 1:**
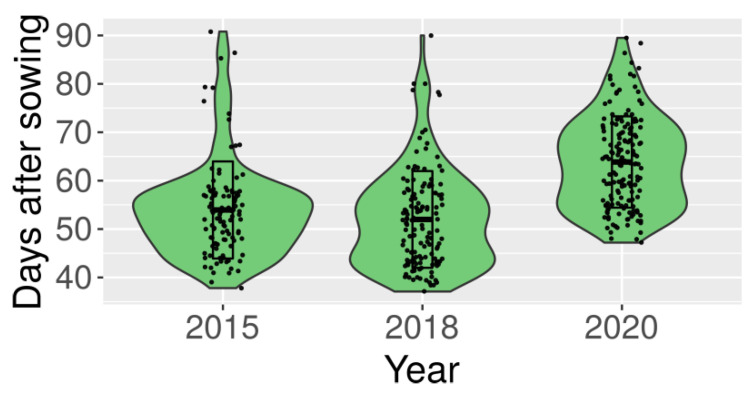
Distribution of the mean number of days from sowing to flowering in white lupin germplasm collection in years 2015, 2018 and 2020. Plants were cultivated in greenhouse without pre-sowing vernalization. Rectangles visualize mean and standard deviation values.

A white lupin germplasm collection (Supplementary Table S1) was phenotyped in greenhouse conditions for time from sowing to flowering without vernalization in three years, 2015, 2018 and 2020. In two years—2015 and 2018—vernalization responsiveness was evaluated as well. Observation data is provided in Supplementary Table S2, whereas calculated mean values with standard deviation are given in Supplementary Table S3. The number of days from sowing to flowering in the absence of vernalization revealed continuous distribution with two major peaks and high variability between genotypes (Figure 1). Observed values ranged from 37.8 ± 0.4 to 90.8 ± 6.5 in 2015 (mean value of 53.9), from 37.1 ± 2.3 to 90.0 ± 0.0 in 2018 (mean value of 52.0) and from 47.2 ± 2.1 to 89.5 ± 1.5 in 2020 (mean value of 63.9).

The general trend of observed variation in time to flowering followed differences in domestication status (Table 1). Improved germplasm (cultivars and cross derivatives) flowered about 3.9–11.1 days earlier than landraces and wild or primitive lines. Mutants were revealed to flower 13.4–18.6 days later than domesticated accessions. Vernalization response was the highest in mutants (−18.3 ± 13.3 and −20.2 ± 13.3 days in 2015 and 2018 experiments, respectively), intermediate in wild/primitive germplasm (−9.8 ± 6.2 and −15.0 ± 7.4) and in landraces (−7.6 ± 6.9 and −14.4 ± 9.6), whereas the lowest in cultivars (−5.2 ± 5.9 and −6.6 ± 3.9) and in cross derivatives (−3.1 ± 2.0 and −7.4 ± 4.1). Interestingly, three primitive accessions from Turkey, namely 95523 “FRA6708B”, 95524 “FRA6712B” and 95525 “FRA6713B”, annotated in Polish database as “wild or primitive” and in Australian database as “landraces”, revealed the earliest time to flowering and full thermoneutrality. Such an observation indicates that early flowering based on thermoneutrality have putatively appeared in white lupin also in primitive germplasm, independently to selective breeding during recent domestication process.

### 2.2. Flowering Induction in White Lupin Germplasm Collection Is under Polygenic Control

In this study, 17 new PCR-based markers were developed using GBS reads aligned to white lupin transcriptome [26,33] and genome [27] assemblies. These markers originated from the flowering time QTLs located in the linkage group ALB02 at position around 100.2/100.5 cM (QTL2, 6 markers), the linkage group ALB13 around 81.0/84.1 cM (QTL3, 4 markers) and 96.2/99.3 cM (QTL4, 3 markers), and the linkage group ALB16 at 0.9/2.2 cM (QTL5, 4 markers). Taking into consideration detection methods, the were 11 CAPS and 6 dCAPS markers developed (Table 2).

**Figure 2:**
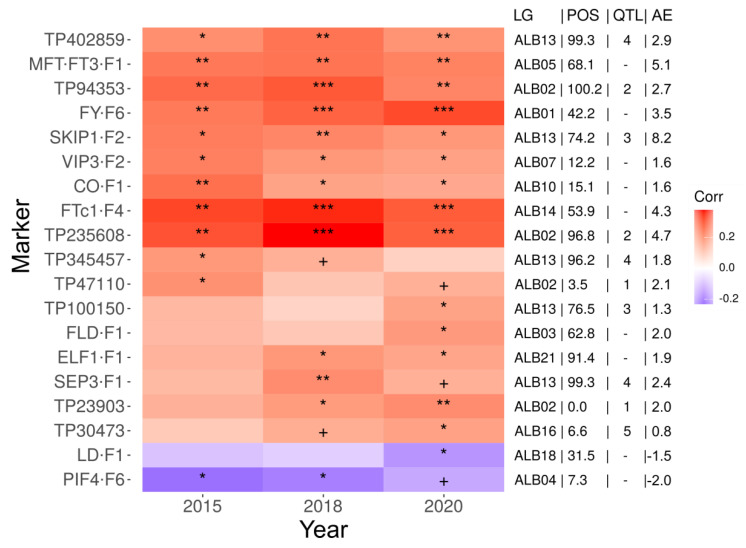
Correlation between the phenotype (the number of days from sowing to flowering) and the genotype (marker segregation) in white lupin germplasm collection. Color scale visualizes Spearman’s rank correlation coefficient values (Corr). Abbreviations are as follows: LG, linkage group; POS; position in the linkage group in centimorgans; QTL, the number of assigned QTL; AE, additive effect (See Supplementary Table S4). *** *p* value <0.001; ** *p* value < 0.01; * *p* value < 0.05; + *p* value < 0.1.

Together with the previously published markers [26,29,30], the final set used for genotyping of white lupin germplasm collection consisted of 50 markers which enabled us to track all major QTLs of flowering time and 29 white lupin homologs of flowering induction pathway genes (Supplementary Tables S4 and S5). Novel alleles (not present in the parental lines of mapping population) were identified for markers FTc1-F4 (16 lines), CO-F1 (2 lines), TP56963 (two lines), MFT-FT3-F1 (one line), and TP114357 (one line). All markers were polymorphic in the analyzed white lupin germplasm collection, however, minor allele frequency (MAF) varied from 0.6% to 48.8% (Supplementary Table S6). Twenty four markers had MAF below 25%, including six markers with MAF below 5% (FRI-F1, ESD4-F8, FTa1-F2, ESD4-F7, SKIP1-F2, and FKF-F2M). Interestingly, two of these markers, FTa1-F2 and SKIP1-F2, were localized in two major QTLs of white lupin flowering time. Moreover, in markers with MAF up to 25.2%, minor alleles were always originating from the P27174 line. Indeed, in case of several markers, very low frequency of wild allele could make it statistically impossible to find significant association between the genotype and the phenotype in germplasm collection despite differences between flowering time in lines carrying opposite alleles. Nevertheless, an analysis of marker polymorphism pattern in germplasm collection revealed the presence of significant correlation between phenotypes and genotypes for 19 markers (Figure 2). This set included markers tagging all major white lupin QTLs of flowering time and 11 homologs of genes involved in regulation of flowering induction, namely *CONSTANS-like* (*CO*, marker CO-F1), *EARLY FLOWERING 1* (*ELF1*, marker ELF1-F1), *FLOWERING LOCUS D* (*FLD*, marker FLD-F1), *FLOWERING LOCUS T* (*FTc1*, marker FTc1-F4), *FY* (marker FY-F6), *LUMINIDEPENDENS* (*LD*, marker LD-F1), *MOTHER OF FT AND TFL1* (*MFT*, marker MFT-FT3-F1), *PHYTOCHROME INTERACTING FACTOR 4* (*PIF4*, marker PIF4-F6), *SEPALLATA 3* (*SEP3*, marker SEP3-F1), *SKI-INTERACTING PROTEIN 1* (*SKIP1*, marker SKIP1-F2), and *VERNALIZATION INDEPENDENCE 3* (*VIP3*, marker VIP3-F2). The highest additive effects were calculated for markers SKIP1-F2 (7.5–9.4), MFT-FT3-F1 (4.0–6.6), TP235608 (3.1–7.9), and FTc1-F4 (3.3–6.2).

### 2.3. Several White Lupin Subpopulations Differring in Flowering Time and Allelic Composition Were Identified

**Figure 3:**
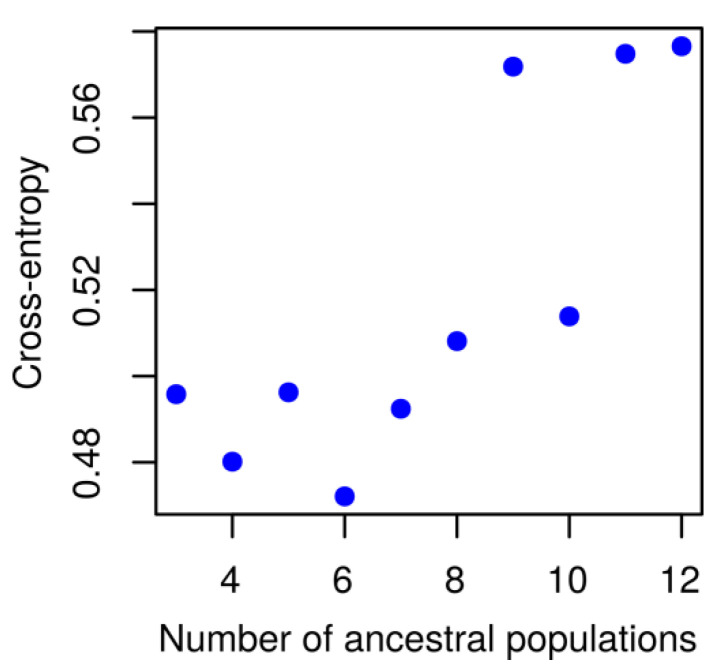
Cross-entropy values obtained for a given number of clusters (ancestral populations).

Data on PCR-based marker polymorphisms obtained for 160 white lupin accessions were exploited for population structure analysis. Minimal cross-entropy values were revealed for six clusters (ancestral populations), followed by a second minimum at four clusters (Figure 3). Based on the results of these calculations, white lupin germplasm collection was divided into six subpopulations. These clusters differed in number of accessions, namely five in group V1, 32 in V2, 20 in V3, 53 in V4, 32 in V5, and 18 in V6 (Supplementary Table S1).

**Figure 4:**
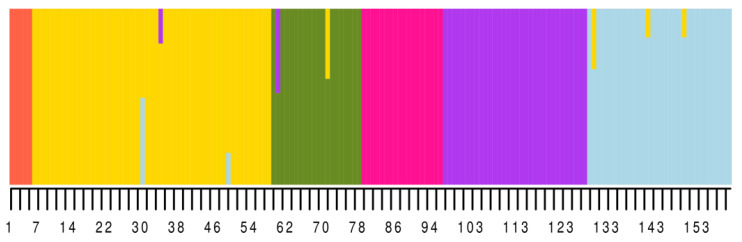
Ancestry matrix constructed for six white lupin subpopulations (*n* = 160).

Molecular analysis of variance (AMOVA) supported the hypothesis that such a grouping into subpopulations is not random (simulated *p*-value: 9.999 × 10^−5^). Membership of all genotypes to six subpopulations were visualized by the ancestry matrix (Figure 4).

**Figure 5:**
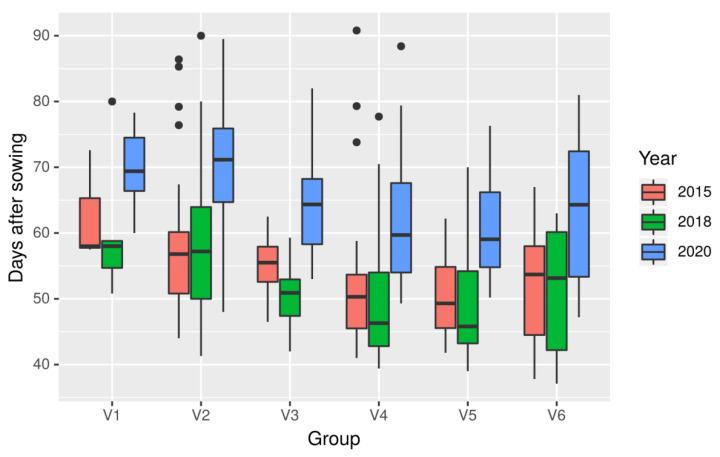
Box plot of days to flowering observed in 6 white lupin subpopulations.

Plants in these subpopulations showed the following mean values of number of days from sowing to flowering: V1, 64.5 ± 9.5 days; V2, 62.8 ± 12.0; V3, 57.7 ± 9.0; V4, 54.9 ± 10.8; V5, 53.9 ± 8.9; and V6, 55.8 ± 11.5 days. There were significant differences between years (*p*-value < 2 × 10^−16^) and groups (*p*-value 3.7 × 10^−11^) and no significant interaction between years and groups (*p*-value 0.996) (Figure 5).

Observed differences in flowering time of early lines between the year 2020 and years 2015 or 2018 may result from the differences in the number of days with maximum temperature above 20 °C recorded during the first 35 days of the experiments, reaching 13 days in 2015, 14 days in 2018 and 4 days in 2020. As the greenhouse was equipped with cooling based only on window opening/closure, high outside temperature directly translated into warmer conditions inside the greenhouse. It is the well-known fact that higher temperature accelerates plant growth and development.

Then, we tested the correlation between distribution of marker polymorphism and observed time to flowering within subpopulations (Supplementary Table S7). Thirteen markers revealed significant correlation (*p* value ≤ 0.05) with time to flowering in at least one year in one group, nine in two groups and two in three or four groups. This set included one marker from the QTL1, four markers from the QTL2, two markers from the QTL3, five markers from the QTL5, and ten markers anchored in sequences of genes which have not been assigned to any of the QTLs.

Taking into consideration the number of years, ten markers revealed significant correlation (*p* value ≤ 0.05) with time to flowering in at least two years within a particular group, including five markers significantly correlated across all three years (Table 3). One marker, TP86766 derived from the QTL5, was significantly correlated with time to flowering in two subpopulations (V4 and V5) across all three years of observations.

Tukey’s tests supported the hypothesis of lack of difference in flowering time between clusters obtained in the population structure analysis for pairs V2-V1, V3-V1, V4-V3, V5-V3, V6-V3, V5-V4, V6-V4, V6-V5, and discarded such a hypothesis for pairs V4-V1, V5-V1, V6-V1, V3-V2, V4-V2, V5-V2, and V6-V2. Therefore, for subpopulation pairs which in Tukey’s test analysis showed a difference in flowering time, χ^2^ analysis was performed to test if differences in allele frequencies are significant or not. Between groups V1 and V4 differences in allele frequencies were significant for 16 markers, between V1 and V5 for 21 markers, between V1 and V6 for 11 markers, between V2 and V3 for 17 markers, between V2 and V4 for 16 markers, between V2 and V5 for 19 markers and between V2 and V6 for nine markers. No marker revealed significant differences in allele frequencies in all (seven) pairwise comparisons, however, two markers (TP402859 and SEP3-F1) showed such differences in six pairs, another two markers (GI-F1 and FLD-F1) in five pairs, and five markers (TP23903, VIP3-F2, TP288840, TP100150, TP3177) in four pairs (Supplementary Table S8). Besides FLD-F1 and VIP3-F2, these markers originated from QTL1, QTL3 and QTL4. Such a result provided additional evidence highlighting hypothetical involvement of genes and QTLs represented by these markers in flowering time control in white lupin.

## 3. Discussion

### 3.1. Contribution of FLOWERING LOCUS T Genes to Early Flowering in White Lupin

In white lupin, early flowering accessions were found in both wild/primitive and domesticated germplasm. Vernalization responsiveness in mapping population, as well as in germplasm collection, was revealed as a continuous trait suggesting quantitative (polygenic) regulation. In the *L. albus* mapping population, several QTL loci for time to flowering were identified hitherto, including five QTLs confirmed in at least two years of observations [24,25,26]. Following the construction of a high-density reference linkage map, sequence-defined markers flanking these QTLs were developed [26,29]. Moreover, recent research highlighted candidate genes for four QTLs, namely *GIGANTEA* (*GI*) for the QTL1, *FLOWERING LOCUS T* homolog a1 (*FTa1*) for the QTL2, *SEPALLATA 3* (*SEP3*) for the QTL4 and *FRIGIDA3* (*FRI3*) for the QTL5 [30]. In this study, white lupin molecular toolbox was supplemented with another 17 CAPS and dCAPS markers to facilitate efficient PCR-based tracking of all five major QTLs in germplasm which is unrelated to mapping population. Thus, this study confirmed the viability of all major white lupin QTLs for time to flowering, highlighted by statistically significant correlations between the phenotype and the genotype identified by particular markers (see Figure 2 and Table 3).

Correlation coefficients revealed in this study as significant reached the maximum values from 0.32 to 0.38 in the whole white lupin germplasm collection and from 0.35 to 0.95 in the particular sub-populations. Such values are typical for multi-locus traits. Indeed, in our previous study, targeting narrow-leafed lupin, a species with vernalization independence conferred by just a single gene [34], the marker anchored in this gene reached correlation coefficient values from −0.48 (yield) to 0.80 (flowering time), whereas PCR-based markers from other genes, also evidenced as statistically significant, reached values from 0.20 to 0.38 [35]. A similar study in soybean revealed, for the marker significantly associated with maturity dates and plant height, correlation coefficient values of −0.27 and −0.51 [36].

Interestingly, only two (*FRI3* and *SEP3*) from four candidate genes (*FRI3*, *FTa1*, *GI*, and *SEP3*) highlighted by a recent mapping population study [30] revealed significant correlations with time to flowering in this study targeting germplasm collection. The lack of statistical support for the *FTa1* from QTL2 gene may result from very low frequency of the wild allele, which was found only in three accessions: two Ethiopian landraces and one Polish breeding line. Moreover, *FTa1* indel marker is localized in the third exon of this gene, whereas major regulatory components of *FT* expression in *Arabidopsis thaliana* were evidenced to be localized in the promoter and in the first intron [37,38,39,40]. Nevertheless, three other markers from QTL2, namely TP235608, TP94353 and TP56963, were found to be significantly correlated with time to flowering in analyzed germplasm (in all years). Contrary to the *FTa1*, the lack of correlation between *GI* marker from QTL1 and flowering time cannot be simply explained by low MAF value, because wild allele was present in about 19% of lines. Two markers flanking QTL1 were significantly correlated with early phenology but only in one or two years.

The present study evidenced also significant correlations between several other genetic components of flowering regulation pathways, including also another white lupin *FT* homolog, an *FTc1* gene –both in the whole analyzed collection as well as in one of the subpopulations (V5). In the narrow-leafed lupin, *Lupinus angustifolius* L., early flowering is based on vernalization independence originating from just two natural mutations (named *Ku* and *Jul*) discovered in domesticated germplasm in 1960s [41,42]. *Ku* is based on a large (1.4 kbp) deletion in the promoter region of one of the four *L. angustifolius FT* homologs, *FTc1* [34]. This deletion carries potential binding sites for several transcription factors acting as *FT* gene repressors in *A. thaliana* [43]. The second *L. angustifolius* domesticated early phenology mutation, *Jul*, was recently revealed to be a 5162 bp deletion in the *FTc1* promoter region, fully encompassing the *Ku* indel [44]. Recently, a fourth *FTc1* allele, *Pal*, was found in a wild population originating from Palestine, carrying 1208 bp deletion partially overlapping with *Ku* [44,45]. Recent gene-based genome-wide association study confirmed that this series of indels in *FTc1* has a major effect on time to flowering and maturity in diversified narrow-leafed germplasm collection [35].

The release of two white lupin high quality genome assemblies [27,28] provided an unprecedented opportunity to search for similar indels in *FT* homologs in this species, both by whole-genome shotgun and PCR-based approaches. Recent comparative mapping approach performed in yellow lupin, *Lupinus luteus* L., revealed that a major QTL for vernalization responsiveness in this species is localized in a linkage map segment syntenic to the narrow-leafed lupin genome region carrying the *FTc1* gene [46,47,48]. All these observations support the conclusion on the highly conserved contribution of the *FTc1* homologs into vernalization pathway in the Old World lupin clade.

The involvement of an *FTc* in flowering time control is relatively rare phenomenon in plants, as *FTc* homologs appeared only in legumes as a result of whole-genome duplication event(s) [43]. Thus, in the legume model plant, *Medicago truncatula* L., vernalization responsiveness is more likely conferred by the *MtFTa1* gene (which is also highly induced by long day conditions), whereas photoperiod pathway by the *MtFTb1* and *MtFTb2* genes [49,50,51]. In pea, *FT* genes showed transcriptional sub-functionalization and one of those, *FTa1*, underlies the pea *GIGAS* locus, essential for flowering under long-days and positive for flowering under short-days [50]. In soybean, which is a vernalization independent species, three genes belonging to *FTa* and *FTc* clades confer just the photoperiod response [52,53,54,55]. In chickpea, altered expression of a cluster of three *FT* genes (*FTa1*, *FTa2* and *FTc*) is associated with early phenology [56], however, a major QTL for vernalization response in this species is localized in a genome region lacking any *FT* homolog [57].

### 3.2. Candidates from Photoperiod, Vernalization, Autonomous and Heat-Responsive Pathways for Flowering Time Control in White Lupin

Besides *FTc1*, our study revealed significant correlations between time to flowering and sequence polymorphism in markers anchored in the sequences of the following genes: *CO-like*, *ELF1*, *FLD*, *FRI*, *FY*, *LD*, *MFT*, *PIF4*, *SEP3*, *SKIP1*, and *VIP3*. Without any data about linkage disequilibrium decay we could not infer about the size of haplotype blocks represented by these markers. Therefore, we focused on the analysis of hypothetical involvement of genes represented by particular markers in regulation of flowering time in white lupin.

CO-F1 marker was found to be significantly correlated with flowering time of white lupin in all three experiments, both in the whole set of lines as well as in one of the subpopulations (V6). *CO* gene is a well-known central regulator of photoperiod pathway in *A. thaliana* [58,59]. In *M. truncatula*, a homolog of this gene was localized in the major QTL for flowering date and revealed different expression profiles in parental lines contrasting for flowering time [60,61]. The association between *CO*-like gene and flowering time was also confirmed for *Medicago sativa* [62]. These observations support the concept of choosing *CO* gene as one of the targets in artificial selection in white lupin.

ELF1-F1 marker revealed significant correlation in two years (2018 and 2020). The lack of statistical support for correlation in the first experiment might be related with the lower number of white lupin lines analyzed, especially those with late flowering phenotype. *ELF1* gene is hypothetically involved in flowering regulation in *A. thaliana*, however, particular mechanism has not been yet deciphered [63]. *ELF1*, together with an *FLD* gene and other six potential flowering time-regulating genes, were highlighted by genome-scale association and QTL mapping of complex flowering time trait in chickpea [64].

FLD-F1 and LD-F1 markers showed significant correlation with flowering time in the last year, whereas FY-F6 marker in all three years. *FLD*, *FY* and *LD* in *A. thaliana* are components of the autonomous pathway that promotes flowering independently of the photoperiod and vernalization [65,66]. It was evidenced that soybean genome encodes a functional copy of an *FLD* gene which promotes flowering when introduced into late flowering *A. thaliana fld* mutant [67].

FRI31-F1 marker revealed significant association with flowering time in one of the subpopulations (V6) in the years 2015 and 2020. Besides the floral repressor *FLOWERING LOCUS C* (*FLC*), *FRI* is a second key component of vernalization pathway in *A. thaliana*, and allelic variation of *FRI* accounts for the majority of natural flowering time variation in germplasm collection of this species [68,69]. *FRI* upregulates expression of the *FLC* by acting in a supercomplex that establishes a local chromosomal environment facilitating production of the *FLC* mRNA [70]. Nevertheless, despite revealed significant association between the *FRI* marker and time to flowering in white lupin, direct translation of this mechanism from *A. thaliana* into this species is not possible, due to the lack of any *FLC* homolog in white lupin and other legume genomes, except soybean [30,71,72]. Interestingly, in soybean, SNP variation in *FRI* sequence was also found to be highly associated with flowering time, shedding new light on potential contribution of this gene into flowering regulation network apart from the vernalization pathway [73].

VIP3-F2 marker, tagging another component of vernalization pathway in *A. thaliana*, was significantly associated with white lupin flowering time in all experiments. It was revealed that loss-of-function mutation in the *VIP3* gene suppress the effect of *FRI* on *FLC* expression and flowering time in *A. thaliana* [74,75]. To our best knowledge, the present study is the first report on association between *VIP3* and flowering time in legumes.

Similarly to VIP3-F2, SKIP1-F2 marker also revealed significant correlation with white lupin flowering time in all experiments. *SKIP* is a component of flowering regulation network linking alternative splicing and the circadian clock in *A. thaliana* [76]. It works as a splicing factor as part of the spliceosome and as a transcriptional activator interacting with *EARLY FLOWERING 7* (*ELF7*) [77]. *SKIP* is required for the splicing of *serrated leaves and early flowering* (*SEF*) pre-messenger RNA (mRNA) in *A. thaliana* [78]. A *SKIP* homolog in soybean, *GmGBP1*, acts as a positive regulator upstream of *GmFT2a* and *GmFT5a* and promotes flowering on short days. Moreover, natural variation in *GmGBP1* promoter sequence is associated with photoperiod control of soybean flowering time and maturity [79]. Transgenic *A. thaliana* with the ectopic overexpression of *GmGBP* revealed advanced flowering under long days via photoperiodic and gibberellin pathways (including *CO* and *FT* genes) and delayed flowering under short days via autonomous pathway (*SVP* and *FLC*) [80].

PIF4-F6 marker was significantly correlated with white lupin flowering time in two years (both in the whole set of lines and in the subpopulation V2). *PIF4* is associated in variation of ecologically important traits in *A. thaliana*, including time to flowering [81]. Expression of *PIF4* is induced proportionally to the increase of ambient temperature (at least in the range 12–27 °C), resulting in strong induction of the *FT* gene and overcome of late flowering phenotype of *A. thaliana* [82]. Similar correlation of PIF4 expression and temperature was also observed in soybean [83]. In our study, *PIF4* marker revealed significant association with flowering time in white lupin collection in 2015 and 2018, and non-significant in 2020. Indeed, the number of days with temperature above 20 °C during first 40 days of the experiment was higher in 2015 and 2018 years than in 2020 (14 and 17 vs. 7). Such an observation may explain observed differences in correlation values and provide some support on the conserved function of *PIF4* as a thermosensory activator of flowering in white lupin.

Two *MFT* markers, MFTa1-F1 and MFT-FT3-F1) showed significant correlation with white lupin flowering time in all years, one in the whole collection and the second in the subpopulation V6 (together with *CO* and *FRI*). *MFT* genes constitute the basal clade among phosphatidylethanolamine-binding (PEPB) domain genes and are present in angiosperms, gymnosperms, lycophytes and bryophytes [84]. Duplication of an ancient *MFT*-like gene hypothetically contributed to the radiation of seed plants [85]. Overexpression of the *MFT* gene in *A. thaliana* resulted in slightly early flowering under long days [86]. It was recently evidenced that the *MFT* functions as a key *A. thaliana* repressor of germination under far-red light conditions [87]. Moreover, *MFT* is considered a potential candidate gene for a major QTL that alters *A. thaliana* flowering time at elevated CO_2_ [88]. CO_2_ concentration in greenhouse at seedling stage can be higher than outside due to the vigorous soil respiration and lower canopy photosynthetic rate [89]. Thus, any of the mentioned functions could be assigned to explain observed significant correlation between white lupin flowering time and *MFT* gene-based marker polymorphism.

### 3.3. Perspectives for Molecular-Assisted Breeding of White Lupin

High number of markers which significantly correlated with flowering time suggests that genome-wide selection should be a method of choice for white lupin breeders, rather than the classical approach utilizing marker-assisted selection. Introduction of preferred alleles on one-by-one basis can be inefficient, due to epistatic interactions between major components of flowering regulatory network [90]. Phenotyping studies revealed that white lupin germplasm collection has variability of many agronomic traits, including, besides time to flowering, winter survival, pod fertility, pod wall proportion, number of leaves on the main stem, several yield related traits and anthracnose resistance [11,12,13,29,91,92,93,94]. Despite relatively small genotype sample size (83 landraces and eight varieties), genomic prediction for grain yield, winter plant survival and onset of flowering provided prospective output, manifested by model-based predictive ability values as high as 0.84–0.86 [10]. In that study two models were tested, Bayesian Lasso [95] and ridge regression best linear unbiased prediction (rrBLUB) [96,97], providing similar results. Genomic prediction of grain yield in three European sites with contrasting climate (Mediterranean, subcontinental or oceanic) displayed relatively high intra-environment (up to 0.71) and cross-environment (up to 0.51) predictive abilities, providing economic justification for genomic selection strategy in white lupin breeding [11].

### 3.4. Concluding Remarks

This study provided novel evidence for quantitative control of flowering time in white lupin by supporting all major QTLs derived from mapping population studies as well as designating new candidate genes from major molecular pathways regulating flowering induction in plants. A very high majority of markers revealed an association of a Kiev Mutant allele with accelerated flowering, indicating that desired alleles have been already introduced into domesticated germplasm. Nevertheless, two important components of flowering regulatory network, *PIF4* and *LD*, showed an opposite association, opening up the development potential for white lupin breeders. Taking into consideration the results of studies obtained for other legume species, such as narrow-leafed lupin or soybean [34,79,98], resequencing of candidate genes in wider germplasm background, including also promoter regions, could be valuable by providing novel alleles conferring early or intermediate phenology.

## 4. Materials and Methods

### 4.1. Plant Material

The set of 160 *L. albus* lines, derived from the European Lupin Gene Resources Database maintained by Poznań Plant Breeders Ltd. station located in Wiatrowo as well as from the National Centre for Plant Genetic Resources: Polish Genebank, Plant Breeding and Acclimatization Institute–National Research Institute, Radzików, 05–870 Błonie, Poland, was used in the study. This germplasm collection contained 63 wild or primitive populations, 51 landraces, 31 cultivars, 12 cross derivatives and three mutants. These lines originated from 23 countries. The information on germplasm donor, country of origin and domestication status was provided in Supplementary Table S1.

### 4.2. Profiling of Time to Flowering and Vernalization Responsiveness in Controlled Environment

Phenotyping experiments were performed in a greenhouse located at the Institute of Plant Genetics, Polish Academy of Sciences, Poznań, Poland (52°26′ N 16°54′ E) during growing seasons of 2015 (sowing date 10.04), 2018 (sowing date 22.03) and 2020 (sowing date 19.03) under ambient long day photoperiod (12–17 h). To randomize greenhouse-related effects between years and lines, the experiment was performed every year in a different greenhouse and in a different design (Supplementary Figure S1). Automatic heating was used to keep the minimum air temperature above 18 °C. Cooling was maintained by temperature-dependent ventilation system (activated at 22 °C). Vernalization was carried out by placing seeds for 23 days at 5 °C in darkness on moist filter paper in Petri dishes. Non-vernalized plants were sown five days before the end of vernalization period and cultivated at ~23 °C to keep a similar thermal time [99]. Time to flowering was recorded as the number of days from sowing of vernalized plants until the first fully colored petal was developed. Observations were made for each plant separately. The average number of plants with observations in 2015 was 5.1 for non-vernalized variant (min 3, max 9, *n* = 105) and 4.6 for vernalized variant (min 3, max 8, *n* = 104), in 2018 it was 7.9 for non-vernalized variant (min 3, max 10, *n* = 140) and 9.0 for vernalized variant (min 3, max 10, *n* = 138) whereas in 2020 it was 8.8 in non-vernalized variant (min 3, max 10, *n* = 160). Daily mean and maximum air temperature and daily sunshine hours recorded by the nearby localized meteorological station (Poznań-Ławica, 5.1 km away) and theoretical photoperiod hours calculated for this latitude (covering 100 days from sowing date) were provided for reference in Supplementary Table S9.

### 4.3. Development of Molecular Markers for Flowering Time QTLs

PCR-based molecular markers were developed for single nucleotide polymorphisms (SNPs) localized on the white lupin linkage map in the proximity of all major QTLs for flowering time. The following QTLs [26,30] were explored: linkage group ALB02 (QTL1, position around 2.2 cM), ALB02 (QTL2, 100.2/100.5 cM), ALB13 (QTL3, 81.0/84.1 cM and QTL4, 96.2/99.3 cM), and ALB16 (QTL5, 0.9/2.2 cM). Genotyping-by-sequencing (GBS) reads which were previously mapped in these regions, were aligned to transcriptome sequences of parental lines of *L. albus* mapping population [26] as well as the reference transcriptome of *L. albus* roots and leaves (https://legumeinfo.org/data/public/Lupinus_albus/ (accessed date 7 April 2021), gene index LAGI01) [33]. BLASTn algorithm [100] implemented in Geneious v8.1 (Biomatters, New Zealandf) [101] was used for this alignment with max 2 mismatches allowed per sequence.

To map intron/exon boundaries, matched transcript sequences were extracted and re-aligned to the genome scaffolds [27] using progressive Mauve algorithm with gapped aligner MUSCLE 3.6 [102,103]. Mauve alignments consisting of GBS reads, Kiev Mutant, P27174 and LAGI01 transcripts, and genome scaffolds, were screened for the presence of polymorphic loci. The primers flanking these loci were designed using Primer3Plus [104,105]. Depending on the availability of restriction enzymes, SNPs were transformed the cleaved amplified polymorphic sequence (CAPS) [106] or derived CAPS (dCAPS) [107] markers. Restriction sites and dCAPS primers were identified using dCAPS Finder 2.0 and SNP2dCAPS [108,109].

### 4.4. Genotyping of White Lupin Germplasm with PCR-Based Markers

The white lupin germplasm collection which was subjected to phenotyping of flowering time and vernalization responsiveness, was also genotyped with PCR-based markers. The set of markers included markers for flowering time QTLs developed in this study as well as previously published markers designed for white lupin homologs of flowering control genes [30] and those developed for the anthracnose resistance QTL localized at the linkage group ALB02 overlapping with the flowering time QTL1 [29]. Young 5 week-old leaves were collected from plants cultivated in greenhouse and immediately frozen under liquid nitrogen. Frozen plant tissue (50–100 mg) was homogenized using TissueLyser II (Qiagen, Hilden, Germany) and two stainless steel beads (ø 5 mm) placed in a 2 mL tube (Eppendorf, Hamburg, Germany). DNA isolation was performed using DNeasy Plant Mini Kit (Qiagen). PCR products were amplified using GoTaq G2 Flexi DNA Polymerase (Promega, Mannheim, Germany). Restriction enzyme digestion was performed for 12 h in 20 µL of mixture including 5 µL of post-PCR mixture, one unit of enzyme and appropriate amount of water and buffer to reach concentration recommended by the enzyme manufacturer. Restriction products were separated by agarose gel electrophoresis, with the agarose concentration (1–3%) adjusted to follow the size of the expected digestion products. Wide range agarose (Serva, Heidelberg, Germany) was used for most applications, however, markers yielding polymorphic cut products shorter than 100 bp were resolved using high resolution 3:1 agarose (Serva).

The list of markers used in the study with localization on linkage map, QTL assignment, accession numbers and sequences of both alleles is provided in Supplementary Table S4. Information on PCR annealing temperature, polymorphism detection method, expected products and agarose gel concentration is provided in Supplementary Table S5. Two biological replicates were analyzed per line. Kiev Mutant marker alleles were assigned “1”, P27174 marker alleles were assigned “2”, whereas heterozygotes “1.5”. Additional alleles (different than those observed in Kiev Mutant or P27174) were assigned “C” or “3”.

### 4.5. Population Structure Analysis

Population structure analysis was performed with LEA R package [110]. Obtained marker data were converted to multi-allele geno format using a custom Python script converting every allele into a specific character symbol. Next, the optimal number of clusters (k) in the investigated population was determined using admixture analysis [111,112] with snmf function from the same package. The entropy criterion evaluating the quality fit of the statistical model to the data was calculated in 1000 repetitions and 10,000 iterations. The optimal number of clusters was set as k with minimal cross-entropy values [113,114]. The ancestry matrix for optimal k = 6 was visualized with the barchar function from LEA package.

### 4.6. Statistical Analysis

All statistical analysis were preferred in R software (R Core Team 2013, Vienna, Austria) using base function. Simple regression was used to estimate QTL/marker additive effects. The correlation between alleles in all 50 loci and flowering time was calculated using Spearman’s rank method [115]. The calculation was made for the whole population and separately for each cluster obtained by population structure analysis. Homozygote wild (P27174) got rank 3, homozygote cultivated (Kiev Mutant)–rank 1, and heterozygote–rank 2. Novel alleles, which occurred only for some markers and few lines, were treated as missing values. Giving rank 1 or rank 3 for novel alleles had negligible effect on obtained correlation values, highlighted by ~0.99 correlation of the results. The two-way variance analysis was performed to test the hypothesis of lack of difference in flowering time between clusters obtained in the population structure analysis. The variance model was given by the following formula:

$$Y_{ijk}=\mu +\alpha_{i}+\beta_{j}+\epsilon_{ijk}$$

where $Y_{jjk}$ is flowering time of k-th genotype in j-th year and i-th cluster, $\alpha_{i}$ is i-th cluster, βj is j-th year, *µ* is cluster × year interaction and $\epsilon_{ijk}$ is the random effect component. The true difference between the mean was tested using Tukey’s ‘Honest Significant Difference’ method [116]. The $\chi^{2}$ test was used to test the hypothesis of lack of difference in allele frequency between clusters, which in ANOVA analysis showed a difference in flowering time.

## Figures and Tables

**Table 1:** Mean number of days from sowing to flowering observed in white lupin germplasm in years 2015, 2018 and 2020.

Type	2020 n ^1^	2018 n	2018 v ^2^	2015 n	2015 v
wild or primitive	67.4 ± 8.6 ^3^	54.3 ± 8.2	39.4 ± 2.7	56.6 ± 8.8	46.8 ± 6.2
landrace	64.7 ± 9.3	53.5 ± 11.3	38.8 ± 2.8	53.6 ± 9.4	46.0 ± 4.3
mutant	74.5 ± 8.6	59.5 ± 14.4	39.4 ± 1.5	65.1 ± 15.5	46.8 ± 2.3
cultivar	56.3 ± 6.2	44.5 ± 6.6	38.0 ± 5.5	49.7 ± 10.4	42.7 ± 2.8
cross derivative	58.5 ± 5.8	46.1 ± 5.1	38.7 ± 3.2	46.4 ± 3.6	43.3 ± 2.1

^1^ Non-vernalized plants; ^2^ Vernalized plants; ^3^ Standard deviation.

**Table 2:** New molecular markers developed in this study to supplement marker-assisted tracking of flowering time QTLs in white lupin germplasm collection.

Marker Name	QTL	Primers (5′→3′)	Detection Method	Products Kiev Mutant (bp)	Products P27174 (bp)
TP56963	2	TGCTCGAAATGCCCAAATCCATCA TTGATGCTCGCAGTGAAGAGATAA	CAPS, *Hin*fI	878, 384	878, 304, 80
TP235608	2	GTAGTCCCAAACATGAACGCAG TCATCTGCATACTTGTCATTCCT	CAPS, *Afl*II	217	179.38
TP94353	2	CAGCATTTATGTTGTTGGGACA GGAACCCTGCAATTTGGATAAGG	CAPS, *Rsa*I	60, 51	111
TP278885	2	CCATTTGAATAGCTGCAAATCGCTTCCG CCTTTGATTGTTGAAGCCTATGC	dCAPS, *Hpa*II	112	86, 26
TP115697	2	TGGCTCCTGTTATGTCACTCA TGAATTTGAGACAAACTCAGTGGTA	dCAPS, *Rsa*I	111, 24	135
TP114357	2	GCCATTCTGGATGGATAACCG TGGACCATCAGCTGACTTCAA	dCAPS, *Hpa*II	124	105, 19
TP100150	3	TATTGCAGCCAATCCATCACTC ACTTTCTTCATCTGATGTTGACGA	CAPS, *Hpy*Ch4V	87, 38, 30, 5	117, 38, 5
TP288840	3	CTGCAATATATTCTTTAAGACCTGAT CTGGAGGAATCTAATATAAGTTGTT	dCAPS, *Mbo*I	60	37, 23
TP3177	3	CGTGACAAGTGTTCCACGG ATCTGGTTGGAAGCTTGTTGTG	CAPS, *Ssp*I	169	114, 55
TP360542	3	GAGCCAGGAATAAGGGTGGTG ACTGGATAGTAAAACCCCATAGAATTACT	dCAPS, *Taq*I	113	82, 31
TP345457	4	CACAATTCACTACCACAGATCAACC GATTTCGTCCATCCAAGGATTCTTC	CAPS, *Bse*DI	227, 39, 12	143, 84, 39, 12
TP11750	4	AAAACCACTGAAAAGGTTCCACA CAGGCGATAATATACTCGTCCA	CAPS, *Aci*I	209	135, 74
TP402859	4	CTGGTGGCAAAAGAAGCAGAA AAAGCCAGGAAAGCACATTGG	CAPS, *Hpa*II	198	112, 86
TP2488	5	ACCTTGTTATTGATGCTAGCTTCT TGTTTGAGGGAAGGCAGGTGGAAT	dCAPS, *TspEI*	48, 25, 21	48, 46
TP86766	5	CAGCATGCAAGAAAGCTG TCCTTTCTTCTCCTTCTCTTTC	CAPS, *Dde*I	64	48, 16
TP47264	5	TAACATGCAGCACTCACCAAC TCTGGTTTCTGGGTAATGAGGA	CAPS, *Mbo*I	171	105, 66
TP30473	5	CAGCACACAACCGCAATAAC ATAATTACAGGAAAATATGGTCTTG	CAPS, *Hpy*CH4V	28.25	53

**Table 3:** Correlation between the phenotype (the number of days from sowing to flowering) and the genotype (marker segregation) in subpopulations of white lupin germplasm collection.

Cluster	Marker	Linkage Group	Position (cM)	QTL	2015 *p*-Value ^1^	2018 *p*-Value	2020 *p*-Value
V2	TP56963 ^2^	ALB02	96.4	2	0.048 ^3^	0.021	0.017
V2	TP235608	ALB02	96.8	2	0.111	0.048	0.018
V2	PIF4-F6	ALB04	7.3	-	0.073	0.044	0.017
V4	TP86766	ALB16	2.2	5	0.046	2.8 × 10^−4^	4.0 × 10^−5^
V4	TP30473	ALB16	6.7	5	0.044	0.019	0.003
V5	FTc1-F4	ALB14	53.9	-	0.051	0.016	0.008
V5	TP86766	ALB16	2.2	5	0.034	0.020	0.003
V6	MFTa1-F1	ALB05	68.1	-	0.039	0.032	0.023
V6	CO-F1	ALB10	15.1	-	0.027	0.009	0.012
V6	FRI31-F1	ALB16	5.8	5	0.003	0.087	0.016
V6	TP2488	ALB16	0.3	5	0.062	0.029	0.039

^1^*p*-value of Spearman’s rank correlation coefficient; ^2^ Only markers showing significant correlation with time to flowering in at least two years within a particular group were listed here; ^3^ Color scale was used to highlight the statistical significance of obtained values as follows: red, *p* ≤ 0.0001; orange, *p* ≤ 0.001; yellow, *p* ≤ 0.01; green, *p* ≤ 0.05; white, *p* > 0.05.

## Data Availability

All data generated in this study are included in this published article and its supplementary information files.

## Supplementary Materials

The following are available online at https://www.mdpi.com/article/10.3390/ijms22083856/s1, Table S1: White lupin lines used in the study, Table S2: Number of days from sowing to flowering observed for white lupin lines used in the study, Table S3: Mean values and standard deviation of flowering time observed for white lupin lines used in the study, Table S4: The list of markers used in the study with localization on linkage map, QTL assignment, accession numbers and sequences of both alleles, Table S5: PCR annealing temperature, polymorphism detection method, expected products and agarose gel concentration for markers used in the study, Table S6: Results of genotyping of white lupin germplasm collection with PCR-based markers, Table S7: *p*-values of Spearman’s rank correlation coefficient calculated for flowering time and marker distribution in white lupin germplasm collection, Table S8: *p*-values of Chi-square test of differences in allele frequencies between subpopulations, Table S9: Meteorological conditions recorded by the nearby localized meteorological station (Poznań-Ławica, 5.1 km) and theoretical photoperiod hours calculated for this latitude (covering 100 days from sowing date). Figure S1. Orientation of greenhouses and arrangement of tables in plant phenotyping experiments.
